# The effects of ketoprofen and meloxicam on oxidative stress through the glutathione pathway after ketamine-xylazine anesthesia and ulcer induction in rats: A comparative study

**DOI:** 10.1016/j.vas.2024.100377

**Published:** 2024-07-14

**Authors:** Azin Sheverini, Ali Khezrian, Ali Shojaeian

**Affiliations:** a Department of Clinical Sciences, Faculty of Veterinary Medicine, Razi University, Kermanshah , Iran; b Research Center for Molecular Medicine, Hamadan University of Medical Sciences, Hamadan, Iran

**Keywords:** Anesthesia, Rat, Oxidative stress, Ulcer induction, Glutathione, Ketoprofen, Meloxicam

## Abstract

Given that oxidative stress (OS) occurs as one of the complications of general anesthesia and surgical procedures, practicing the best and safest anesthesia regimen can have a significant share in various surgeries. So, this study compared the effects of non-steroidal anti-inflammatory drugs (NSAIDs) such as ketoprofen (KTP) and meloxicam (MLX) on OS through the glutathione pathway after the ketamine-xylazine (K-X) anesthesia and ulcer induction in rats to suggest post-operative regimens with promising analgesic and anti-inflammatory effects.

80 healthy adult male Wistar rats, were examined in this study. To obtain the baseline value cardiac blood collected of five rats, and the remaining 75 animals were randomized into three groups of 25, including (i) the control group receiving physiological serum, (ii) the experimental group 1 taking KTP, (iii) the experimental group 2, administered by MLX and all three groups received K-X combination IP after 30 min. Then, a full-thickness ulcer was induced under standard conditions, and the blood samples were collected from groups at T0, T30m, T60m, T24h, and T48h. The serum levels of the desired markers were measured. The study results revealed that the administration of K-X as an anesthetic agent made some changes in the markers of the OS-related glutathione (GSH) pathway. Moreover, KTP and MLX, significantly (*p* < 0.05) augmented the reduced GSH (rGSH), lowered the GSSG, increased the total values of the glutathione disulfide (GSSG) and the rGSH, reduced the rGSH/GSSG ratio, and accelerated the glutathione peroxidase (GPx) activity, but they had high inhibitory effects on the glutathione reductase (GR). Accordingly, both drugs could maintain the balance between the OS markers, caused by general anesthesia. In general, KTP can be a suitable regimen in surgeries wherein analgesia is of importance for less than 24 h, but MLX can be a preferable option if longer analgesia is needed for more than 24 h.

## Introduction

Reactive oxygen species (ROS) are typically produced all through oxygen metabolism in the mitochondrial respiratory chain, the hemoglobin oxidation (autooxidation) reactions, and the respiratory burst of granulocytes in phagocytosis. Multiple external factors, including increased radiation exposure, relative hypertension and hypotension, and some drugs and chemicals have been documented to act as the main sources of the ROS production (Juan et al., 2021). Moreover, postoperative pain has been addressed among the main causes of oxidative stress (OS) (Yilmaz et al., 2014).

Notably, ROS contain oxygen free radicals, such as superoxide anion radical (O_2_^·–^), singlet oxygen (^1^O_2_), hydroxyl radical (^·^OH), and perhydroxyl radical (HO_2_^·^) as well as non-radicals, e.g., hydrogen peroxide (H_2_O_2_), hypochlorous acid (HClO), and ozone (O_3_) (Senoner, Velik-Salchner, Luckner, & Tauber, 2021). At physiological concentrations, ROS are vital for normal cell function, such as oxidation and degradation regulation, defense against invading microorganisms, signal transduction pathways, gene expressions, and cell growth or death regulation, but they cause cell death, apoptosis, and aging at high concentrations owing to their instability and strong tendency to react with other molecules (Juan et al., 2021). Therefore, increased production and/or insufficient performance of antioxidant systems can result in pathological conditions of OS, accompanied by damage or oxidative modifications of some molecules, such as nucleic acids, proteins (or enzymes), and lipids (Pizzino et al., 2017).

Numerous studies have so far investigated the possible factors contributing to higher antioxidant status and lower postoperative OS. As evidenced, the intracellular levels of antioxidants as barriers against OS seriously affect the general anesthesia and surgery outcomes as well as their complications, such as ischemia, trauma, or bleeding (Mantle et al., 2001). There are also some natural protective measures of the body against ROS that are enzymatic, e.g., superoxide dismutase (SOD), catalase (CAT), peroxiredoxin (PRDx), and glutathione peroxidase (GPx), and non-enzymatic, including tocopherol (vitamin E), beta-carotene, ascorbate (vitamin C), and glutathione (GSH) (Senoner, Velik-Salchner, Luckner, & Tauber, 2021). GSH is an intracellular tripeptide thiol in living organisms, which consists of L-cysteine, L-glutamic acid and glycine, and is one of the cellular antioxidants (Kwon et al., 2019).

The synthesis of GSH from glutamate, cysteine, and glycine is sequentially catalyzed by two cytosolic enzymes, γ-glutamylcysteine synthetase (GCS) and GSH synthetase. This pathway is present in almost all cell types, and the liver is the main producer and exporter of GSH. GSH synthesis in animal cells is favored because of the much higher affinity and activity of GSH synthetase (Wu et al., 2004).

Many of the mentioned antioxidants eventually turn into oxidation products that react with glutathione to prevent oxidative stress. Therefore, these antioxidant defense effects of glutathione play an important role in regulating cell proliferation and cell death by mediating the main redox regulatory signaling pathway in the cell. GSH plays this role by removing ROS to thiol-containing cysteine residues in intracellular proteins and repairing damage caused by OS (Won et al., 2015).

As there have been a rising number of surgeries demanding general anesthesia over recent years, practicing anesthesia in a suitable manner seems to be very important to avoid possible complications, given the fact that patients undergoing general anesthesia are exposed to OS before, during, and after surgery (Senoner, Velik-Salchner, Luckner, & Tauber, 2021). The use of analgesics for pain management has also been an integral part of clinical veterinary medicine to improve the conditions in animals. Therefore, various drugs and their combinations have been thus far applied to prevent or minimize pain (Sladky et al., 2000). In this line, non-steroidal anti-inflammatory drugs (NSAIDs) have been commonly utilized in animals thanks to their anti-inflammatory and analgesic effects (Duz et al., 2019).

However, more selective drugs, such as ketoprofen (KTP) or meloxicam (MLX), have shown fewer side effects such as stomach and intestinal ulcers and renal dysfunction compared to non-selective NSAIDs for cyclooxygenase (COX). These selective drugs work by blocking the COX enzyme, which regulates prostaglandin (PGs) production and ultimately reduces pain (Hassan et al., 2016; Raidal et al., 2014). Although MLX is a COX-2 preferential NSAID, KTP is a non-selective COX inhibitor. Evidence further suggests that COX-2 is the predominant COX isoform in the spinal cord, which is associated with pain detection by the central nervous system (CNS) during inflammation (Canduz et al., 2007; Karnik et al., 2006). The results of Edfawy et al.'s study focused on the application of meloxicam liver protection against liver damage caused by CCl-4 and showed that the antioxidant effects, inhibition of free radicals, anti-apoptotic and anti-inflammatory effects of this drug caused the return of superoxide dismutase, catalase, Glutathione-S-transferase and glutathione to normal levels (Edfawy et al., 2012).

From this perspective, preventing intraoperative OS-related damage is of utmost importance to achieve better results in ketamine-xylazine (K-X) anesthesia. Against this background, this study aimed to compare two analgesic agents, here, KTP and MLX, following K-X anesthesia and ulcer induction in rats on the GSH pathway, including reduced GSH (rGSH), GSH oxidized or glutathione disulfide (GSSG), GPx, and glutathione reductase (GR).

## Materials and methods

### Samples

#### Study design

To fulfill this study, 80 healthy adult male Wistar rats, aged five weeks, weighing 250 g (250±20 g) were acquired from the Faculty of Pharmacy affiliated to Kermanshah University of Medical Sciences, Kermanshah, Iran, and then tested in accordance with the guidelines of the Ethics Committee for Animal Experimentation (IR.RAZI.REC.1400-054). Of note, the animals were kept in cages under similar and standard conditions in terms of light, temperature, and humidity with a 12-h day/night cycle and ad libitum feeding. Before performing any injections, five rats were subjected to cardiac blood collection after using inhalation anesthetic ether in order to measure the baseline values of the markers considered. The remaining 75 animals were randomly divided into three groups of 25.

### Used chemicals

Three study groups, including (i) the control group (CG) receiving pure physiological serum subcutaneously (SC) and combined K-X (viz., ketamine 75 mg/kg (Ketamine 10 %, Alfasan, Netherlands) and xylazine 5 mg/kg (Xylazine 2 %, Alfasan, Netherlands)) intraperitoneally (IP) 30 min after the first injection, and no analgesic agents used in this group, (ii) the experimental group 1 (EG1) taking KTP (5 mg/kg) (Ketomax, Rooyan Darou Pharmaceutical Co., Iran) subcutaneously and combined K-X IP after 30 min, and (iii) the experimental group 2 (EG2), administered by MLX (2 mg/kg) (Meloximax Rooyan Darou Pharmaceutical Co., Iran) subcutaneously and combined K-X IP after 30 min (Tavakoli et al., 2012).

### Full-Thickness ulcer induction and blood sampling

**Fig. 1:**
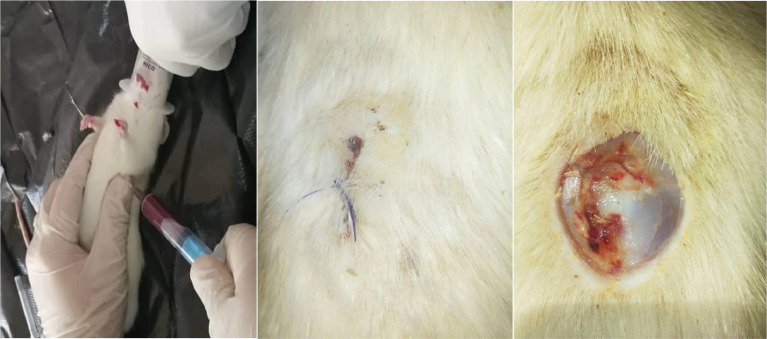
Full-thickness ulcer induction in rats and cardiac blood collection.

After general anesthesia, the rat was positioned in dorsal recumbency and the dorsal surface was disinfected by 70 % ethanol. Utilizing a template with an appropriate measurement index to induce an ulcer at the same distance from the neck in all animals, a circular ulcer with a diameter of 1 cm was then formed via scissors and tweezers. This incision was created in the studied rats to simulate the inflammatory condition in veterinary surgeries. Next, the desired area was checked for bleeding, and subsequently a simple interrupted suture was used to close the incision with 4–0 nylon thread. After that, anesthesia was performed using an overdose of ketamine-xylazine (K-X) compound and cardiac blood samples were collected in compliance with the ethics and easy killing from three EGs with five rats at T0 (before general anesthesia and ulcer induction), and T30m, T60m, T24h, and T48h (i.e., 30 min, 60 min, 24 h, and 48 h after general anesthesia and ulcer induction, respectively) (Masson‐Meyers et al., 2020) (Fig. 1).

### GSH, GSSG, GPx, and GR measurement

The serum levels of the GSH, GSSG, GPx, and GR markers were measured using commercial kits (Novin Navand Salamat Pishtaz Co., Iran). After preparing the samples according to the kit instructions, they were transferred to the wells. Upon performing the steps included, the results were read by the ELISA reader (Bio Tek, Elx800 model, the United States) (Linillos-Pradillo et al., 2023).

### Statistical analysis

The GraphPad Prism (ver. 9) software was used for data analysis. The quantitative data were accordingly reported as mean±standard deviation (SD) with regard to 95 % confidence interval (CI). The normality of the quantitative variables was also determined using the Kolmogorov-Smirnov test and the Shapiro-Wilk test. In case of normality, the comparison of the data in the study groups was done using the two-way repeated measures analysis of variance (ANOVA) and the Tukey's post-hoc test. If there was non-normality, the Kruskal-Wallis test was operated. The significance level of 5 % (p-value<0.05) was also considered for evaluating the intergroup statistical significance.

## Results

At first, the GSH, GSSG, GPx, and GR outcomes were compared between the baseline group (with no intervention) and the EGs at T0 (Table 1).

**Table 1:** Comparing GSH, GSSG, GPx, and GR outcomes in baseline group (baseline: five rats anesthetized with ether and no other intervention) and EGs at T0.

Parameter	Groups	Control	Ketoprofen	Meloxicam
**GSH (µM)**	**Baseline**		10.07±1.81	
	**0min**	10.50±1.66	11.25±0.59	11.10±1.18
**GSSG (µM)**	**Baseline**		0.133±0.019	
	**0min**	0.130±0.022	0.110±0.035	0.098±0.045
**GPX (U/ml)**	**Baseline**		513.80±104.53	
	**0min**	505.30±92.43	496.50±79.10	480.609±97.87
**GR (U/ml)**	**Baseline**		3.99±1.46	
	**0min**	4.07±1.17	5.38±1.02	4.98±1.89

### rGSH

**Fig. 2:**
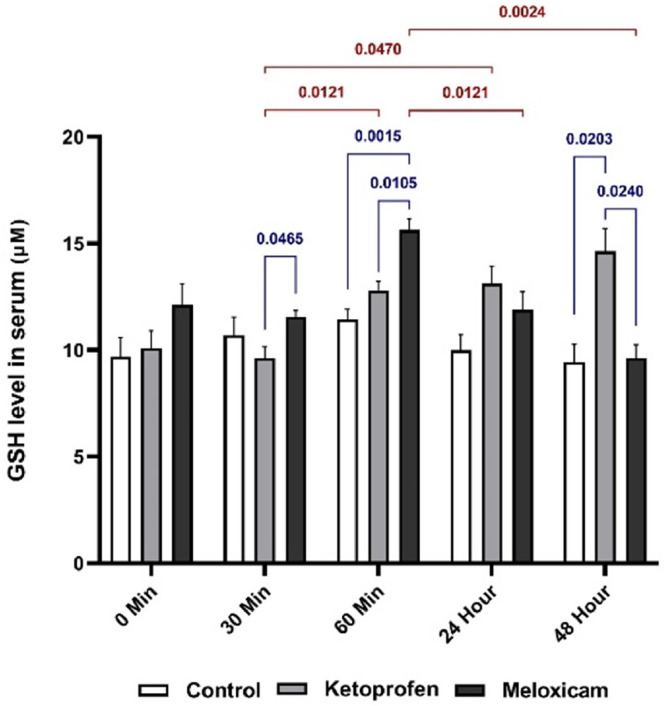
Serum rGSH values in three EGs at five different sampling times The blue is for comparing the results between the study groups at one time, and the red represents the comparison of the outcomes of one EG at different times.

Comparing the rGSH results in the serum of the rats (Fig. 2) indicated a significant difference (*p* < 0.05) between the EGs, receiving KTP and MLX, at T30 and T60m. At both times, the rGSH in the EG1 was lower than that in the EG2, but it was reversed at T48h.

### GSSG

Based on this, the comparison of GSSG outcomes in the serum of rats showed that the level of GSSG in EG2 was higher than in EG1 at T30m and T24h. At T30m, the level of GSSG in CG was higher than that in EG1, and the results comparing T30m with T60m and T24h show an increase in GSSG levels.

### rGSH-GSSG ratio

Comparing the rGSH/GSSG ratio in the serum of the rats (Fig. 4) demonstrated a significant difference between T60m and T0 and T24h (*p* < 0.05) in the CG, and this ratio was higher at T60m than the other two times. In the EG1, a significant difference (*p* < 0.05) was also observed between T30m and T24h, which was associated with a decrease in this ratio. At T60m, there was a significant difference (*p* < 0.05) between the CG and the EG2, and so this ratio was higher in the CG than in the EG2. Moreover, a significant difference (*p* < 0.05) was found between the CG and EG1 at T24h, and this ratio was higher in the EG1 than the CG.

### Total rGSH and GSSG

Fig. 5 depicts the total serum rGSH and GSSG values of three EGs at five sampling times. Comparing the results at T48h displayed a significant difference between the CG and the EG1 (*p* < 0.05). At this time, the rGSH and GSSG levels in the EG1 were higher than those in the CG.

### GPx

Fig. 6 shows the serum GPx activity of the three EGs at different times. As well, Fig. 7 presents the comparison of the activity of this enzyme at different sampling times in each group separately. Examining the GPx activity among different groups at different times revealed a significant difference (*p* < 0.05) between the EG1 and CG and the EG2 at T60m. Besides, there was a significant difference between the EG1 and EG2 at T24h (*p* < 0.05), and the activity of this enzyme was higher in the EG2 than the CG (Fig. 6).

Considering the effects of each drug at different times also demonstrated a significant difference between T0 and T60 or so after general anesthesia in the CG (*p* < 0.05). In the EG1, the activity of this enzyme decreased from that before general anesthesia (T0) to T24h. In the EG2, a significant difference was further seen between the times (*p* < 0.05), and the GPx activity was found to be lower at T60 and T24h than other times (*p* < 0.05) (Fig. 7).

### GR

The study results established that the serum GR activity of three EGs was different at various times and MLX prevented the increase of this enzyme in the early hours, but a significant increase was observed in its activity at T48h compared to that in other groups (*p* < 0.05). Examining the effects of each drug at various times also showed a significant difference between T60 after general anesthesia and T0 in the CG (*p* < 0.05). Moreover, there was a significant rising trend in the serum GR activity in the EG2 at T48h, as compared to other times, except for T24h (*p* < 0.05). The noteworthy point in this Figure was that the GR did not show any significant changes at different times in the EG1 (Fig. 8, Fig. 9).

## Discussion

As the outcome of the imbalance between ROS and inadequate antioxidant systems, OS typically leads to cell damage during general anesthesia (Khoshraftar et al., 2014). All through OS, intracellular GSH content can thus contribute to the damage extent and severity (Park et al., 2018). In view of that, maintaining the balance of OS during general anesthesia and surgical procedures can be useful in the treatment or control of complications. Thus, the present study compared the effects of two NSAIDs as analgesic agents, namely, KTP and MLX, on the intracellular GSH pathway in rats to find the agent that could provide adequate intraoperative protective responses.

The GSH degradation could indicate more oxidation of the cell environment as well as the occurrence of apoptosis. Previous studies have further reflected on this marker as an OS-related factor in the serum level following general anesthesia (Zargari, 2020). Of note, GSH is easily converted into GSSG in reaction with foreign compounds, which may reduce the GSH levels. The GSSG is rapidly regenerated by GR and nicotinamide adenine dinucleotide phosphate (NADPH) or used in the endoplasmic reticulum (ER). Moreover, the GSSG is recycled by protein disulfide isomerase (PDI) and forms GSH. Given these mechanisms, GSH is a very efficient intracellular buffer to deal with OS (Ting et al., 2011).

As concluded in Yakan et al., investigating the effect of MLX on OS in dairy cows subjected to poison modifications, the GSH level had been found to be higher at all times in the analgesia group than in the no-analgesia one. As well, MLX had prevented OS and supported the antioxidant system (Yakan & Duzguner, 2019). In the present study, the GSH level in the EGs receiving KTP and MLX was significantly higher than that in the CG.

In case of increased OS, the GSSG level could multiply; therefore, the proteins containing sensitive and critical thiols in cell signaling, such as receptors, protein kinases, and transcription factors, might undergo some activity changes. In this line, the GSSG could act as a non-specific signaling molecule. The most important buffer for oxidation and degradation regulation as well as cellular regeneration purposes was thus the GSH/GSSG ratio, as an OS marker. In a resting cell, the GSH/GSSG molar ratio could exceed 100/1, while this could decrease to the values of 10/1 and even 1/1 in various models of OS (Zargari, 2020; Zitka et al., 2012).

In the present study, the GSH/GSSG ratio in the CG decreased from 60 min to 24 h, which indicated an increase in OS during general anesthesia without administering any other drugs, but this value in the EGs receiving KTP and MLX maintained a suitable trend at the desired times and showed a significant increase in the EG1 in the final hours, which denoted the OS degradation. Comparing the analgesic effects of metamizole and paracetamol in rats, one study had also found that the GSH/GSSG ratio had elevated, and the OS products had been significantly lower in the groups treated with metamizole, compared to the CGs (Ince et al., 2015). In this study, the GSH/GSSG ratio in the EG1 was significantly higher than that in the CG at 48 h, which was probably due to the growth in the GSH and the fall in the GSSG due to the analgesic effect of this drug.

Besides, GPx could play a leading role in protecting the membrane from damage caused by lipid peroxidation. In a study in 2021 on the GPx activity in freshwater turtles after using inhaled isoflurane anesthesia, a significant increase had been seen in the plasma GP activity three hours after general anesthesia compared to that before anesthesia owing to the changes in blood oxygen concentration (Valčić et al., 2021). In the present study, despite the lack of inhalation anesthesia, a suitable trend was observed in the groups taking analgesics, which showed that the level of this enzyme was maintained in the EG1 up to one hour after general anesthesia and more than that in the CG. In the EG2, there was also the rapid return of the enzyme to the appropriate level, compared to that in the CG at one hour after anesthesia. To clarify the anti-endotoxic effects of pentoxifylline (PTXF), compared to dexamethasone and KTP in sheep endotoxemia, one study had also indicated that the GPx concentration in the KTP-receiving group had been significantly more than other groups after drug administration in all groups at the desired times, except for the negative CG (Shahraki et al., 2016).

**Fig. 3:**
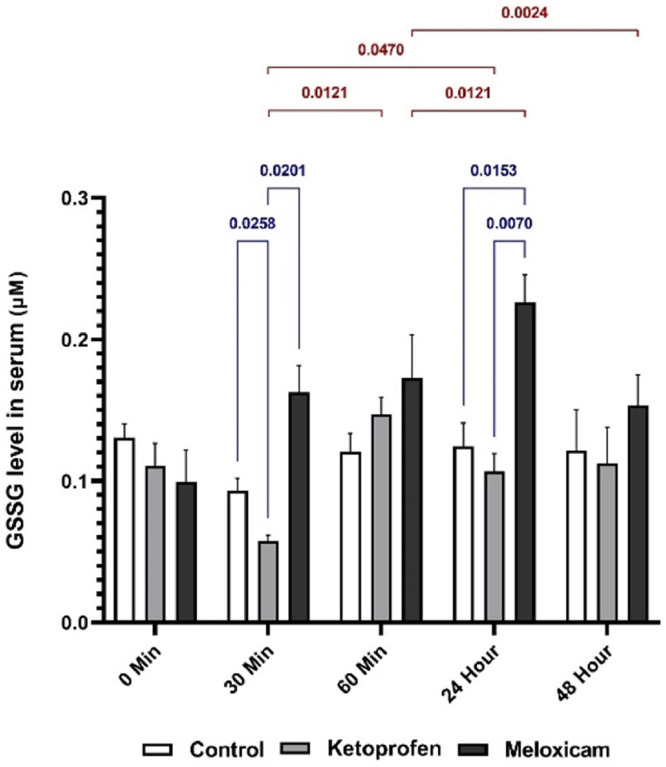
Serum GSSG values in three EGs at five different sampling times The blue is associated with the comparison of the results between the study groups at one time, and the red is for comparing the outcomes of one EG at different times.

The GR also had an indirect effect on oxidative damage prevention by maintaining intracellular rGSH, so its measurement was a marker for OS in the cell (Işık & Soydan, 2023). In a study on the analgesic and anti-inflammatory activity of the aqueous extract of the glycine tomentella root (AGT) in mice, the results had correspondingly shown that the GR and GPx activities had significantly increased in the group treated with AGT. In addition, there was a significant decrease in the OS products, caused by their suppression due to the growth in the GR and GPx activities (Lu et al., 2007). In the present study, the findings established that the GR activity in the EG2 was significantly higher than the CG and the EG1 at T48h after general anesthesia, because this drug accelerated the GR activity as an analgesic according to previous research. Moreover, Nazifi et al. (2019) had investigated the combined effect of tramadol and MLX on OS in dogs and reported that the antioxidant enzymes had significantly decreased, and the OS products had significantly augmented in the tramadol-receiving group, but the group taking MLX had not shown a change in the level of the mentioned cases. In the group treated with MLX together with tramadol, the OS state had also improved by inhibiting lipid peroxidation and boosting antioxidant enzymes. The study results had ultimately indicated that MLX had been effective in reducing OS caused by tramadol (Nazifi et al., 2019). The study outcome adminstrated that use of KTP and MLX reduces pain and inflammation through oxidative stress pathways after surgery, especially complex surgeries such as visceral and orthopedic surgeries. This issue will lead to better patient recovery and increase the quality of ethical considerations in veterinary surgeries (Fig. 3).

**Fig. 4:**
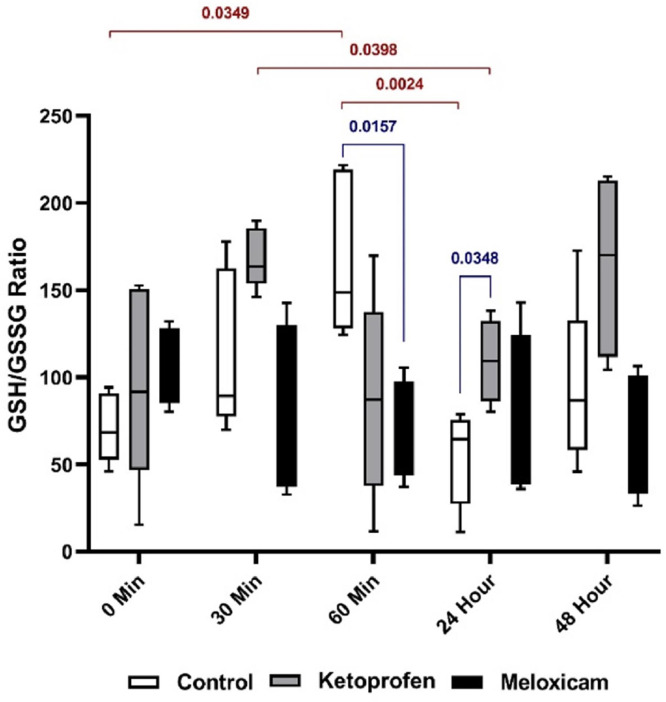
rGSH/GSSG ratio in three EGs at five different sampling times The blue stands for comparing the results between groups at one time, and the red shows the comparison of the outcomes of one EG at different times.

**Fig. 5:**
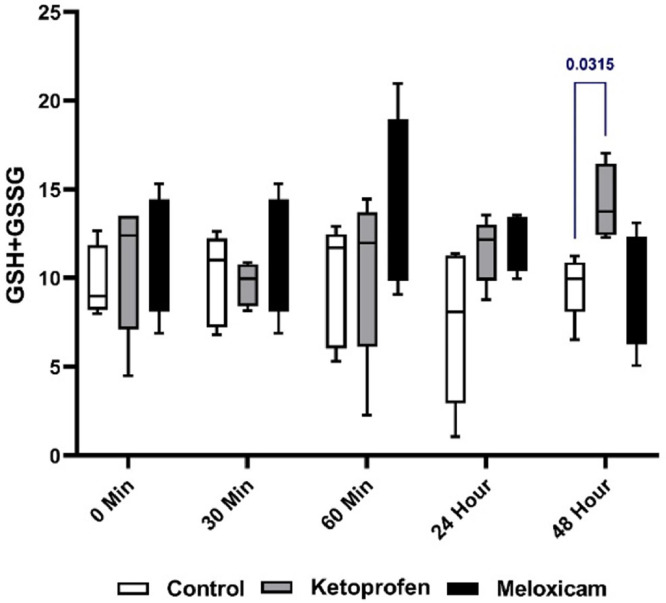
Total rGSH and GSSG in three EGs at five different sampling times The blue is for comparing the results between groups at one time.

**Fig. 6:**
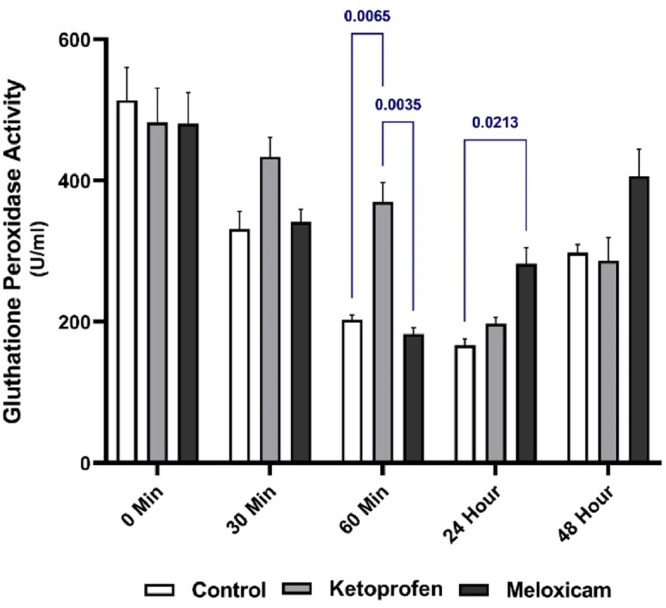
Serum GPx activity in three EGs at five different sampling times The blue is for comparing the results between the groups at one time.

**Fig. 7:**
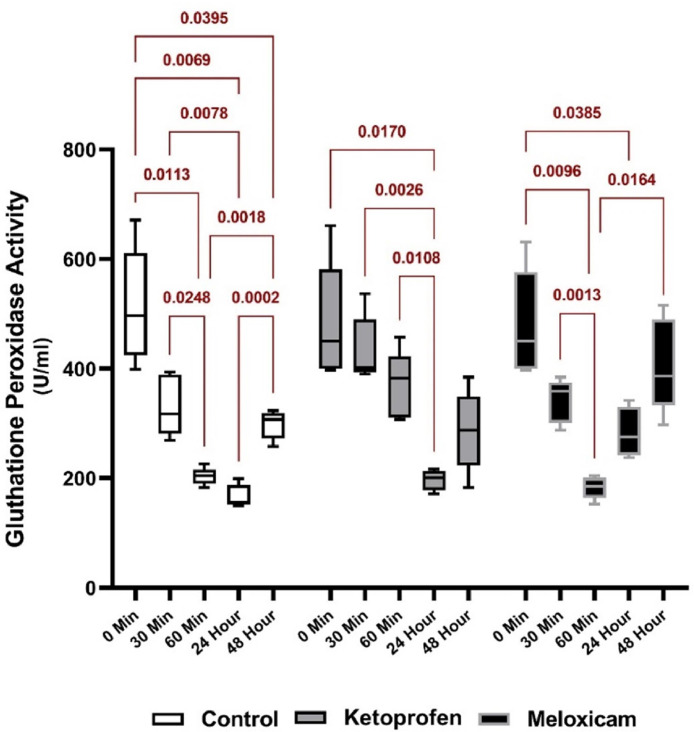
Comparing serum GPx activity at five different sampling times in each EG The red represents the comparison of the outcomes of one EG at different times.

**Fig. 8:**
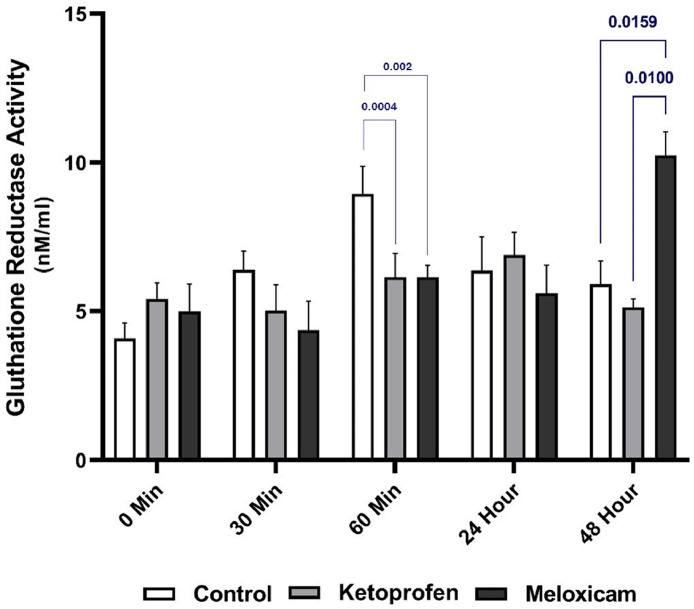
Serum GR activity in three EGs at five different sampling times The blue shows the comparison of the results between groups at one time.

**Fig. 9:**
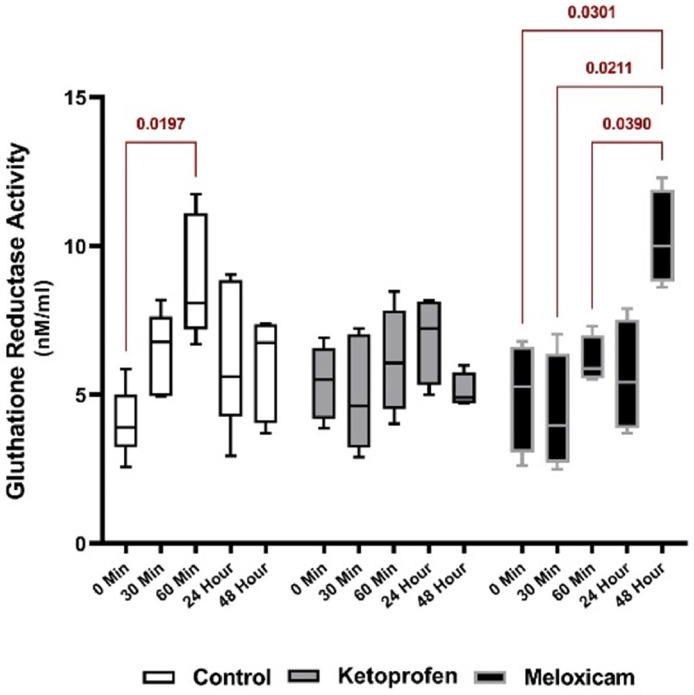
Comparing serum GR activity of each EG at five different sampling times The red color is for comparing the outcomes of one EG at different times.

## Conclusion

Both KTP and MLX as analgesics maintain the balance between OS markers and the GSH pathway after general anesthesia, but the main difference is that the balance is supported by KTP for less than 24 h and MLX maintains the balance longer than 24 h and maintains it better, which could be due to their different half-lives in the body. Considering the increase of antioxidant power and reduction of oxidative stress, probably both drugs can play a role in analgesia. Therefore, KTP may be a suitable regimen in surgeries where analgesia is important for less than 24 h, but if longer analgesia beyond 24 and 48 h is desired, MLX may be a better option. It should be noted that the use of KTP and MLX, no visible side effects were observed in any of the studied groups.

### Abbreviations

OS; Oxidative stress, KTP; Ketoprofen, MLX; Meloxicam, GSH; Glutathione, K-X; Ketamine-Xylazine, ROS; Reactive oxygen species, GPx; Glutathione peroxidase, NSAIDs; Non-steroidal anti-inflammatory drugs, COX; Cyclooxygenase, rGSH; reduced GSH, GSSG; GSH oxidized or glutathione disulfide, GR; Glutathione reductase, CG; Control group, IP; Intraperitoneally, EG; Experimental group, SC; Subcutaneously.

### Ethical statement

No humans were used for studies that are base of this research. All procedures performed on animals were according to The US National Research Council's “Guide for the Care and Use of Laboratory Animals”.

### Financial statement

No grants from funding agencies in the public, commercial, or not-for-profit sectors were received for this research.

### Ethical consideration

No humans were used for studies that are base of this research. All procedures performed on animals were according to The US National Research Council's “Guide for the Care and Use of Laboratory Animals”.

## CRediT authorship contribution statement

**Azin Sheverini:** Writing – original draft, Methodology, Investigation, Conceptualization. **Ali Khezrian:** Writing – original draft, Validation, Methodology, Investigation. **Ali Shojaeian:** Writing – review & editing, Validation, Supervision, Methodology, Investigation, Conceptualization.

## Declaration of competing interest

The authors declare that they have no known competing financial interests or personal relationships that could have appeared to influence the work reported in this paper.
